# Plant MicroRNA Prediction by Supervised Machine Learning Using C5.0 Decision Trees

**DOI:** 10.1155/2012/652979

**Published:** 2012-11-07

**Authors:** Philip H. Williams, Rod Eyles, Georg Weiller

**Affiliations:** Division of Plant Sciences, Research School of Biology, College of Medicine, Biology & Environment, The Australian National University, Canberra, ACT 0200, Australia

## Abstract

MicroRNAs (miRNAs) are nonprotein coding RNAs between 20 and 22 nucleotides long that attenuate protein production. Different types of sequence data are being investigated for novel miRNAs, including genomic and transcriptomic sequences. A variety of machine learning methods have successfully predicted miRNA precursors, mature miRNAs, and other nonprotein coding sequences. MirTools, mirDeep2, and miRanalyzer require “read count” to be included with the input sequences, which restricts their use to deep-sequencing data. Our aim was to train a predictor using a cross-section of different species to accurately predict miRNAs outside the training set. We wanted a system that did not require read-count for prediction and could therefore be applied to short sequences extracted from genomic, EST, or RNA-seq sources. A miRNA-predictive decision-tree model has been developed by supervised machine learning. It only requires that the corresponding genome or transcriptome is available within a sequence window that includes the precursor candidate so that the required sequence features can be collected. Some of the most critical features for training the predictor are the miRNA:miRNA^**∗**^ duplex energy and the number of mismatches in the duplex. We present a cross-species plant miRNA predictor with 84.08% sensitivity and 98.53% specificity based on rigorous testing by leave-one-out validation.

## 1. Introduction

MicroRNAs (miRNAs) are nonprotein coding RNAs of between 20 and 22 nucleotides that attenuate protein production by cleavage, translational inhibition, or sequestering of mRNA in P bodies [1]. They are implicated in several different biological pathways, including plant and animal development, and cancer [2–4]. To better understand the role that miRNAs play in these pathways, large datasets containing RNA-seq, expressed sequence tags (ESTs), and genomic sequences are being investigated for new miRNAs [5, 6]. As these datasets grow in an ever increasing rate, their rapid analysis has become critical. 

Understanding miRNA biogenesis is important when developing predictive models. The mature miRNA originates from an expressed RNA precursor. The precursor folds back to base pair with itself to form a characteristic stem-loop structure. However, not all stem-loop structures are miRNA precursors. The dicer protein cuts a short, double-stranded RNA (miRNA:miRNA* duplex) from the precursor. This double-stranded RNA associates with the RISC complex, where the mature miRNA is retained while the miRNA* is assumed to degrade [7]. The miRNA-loaded RISC complex is responsible for mRNA cleavage, translational inhibition, or sequestering. 

Many methods for predicting miRNAs from sequence data have been reported [6, 8–16]. Some of these methods are directed specifically at ESTs [8] while others are specific for deep-sequencing data [10, 11, 17]. Several methods use machine learning approaches such as support vector machines (SVMs) [6, 9, 14] or decision trees [13, 15]. These methods typically incorporate information based on the predicted minimum free energy (MFE) of the precursor secondary structure. 

Xue et al. [14] used a sliding triplet method to determine characteristic attributes within the triplets of miRNA and non-miRNA stem-loop structures for training an SVM. The authors reported prediction accuracy for miRNA precursors of between 66.7% and 100% on a variety of plant and animal species, as well as viruses after training on known human miRNA precursors.

MiPred employs the random forest machine learning method [13]. It was trained using the same triplet element technique mentioned above, as well as other characteristics including dinucleotide shuffling. The relationship between the MFE of the original sequence and its shuffled counterparts (*Z* statistics) proved to be an important attribute for training this predictor. The MiPred method produced a sensitivity of 95.09% and specificity of 98.21%. Both machine learning techniques described above agree on the high predictive value of specific triplet elements. These are (i) three unpaired bases with a C in the middle position, (ii) three paired bases with a U in the middle, and (iii) three paired bases with an A in the middle.

Approaches designed for deep sequencing data, such as MirTools [10], miRanalyzer [11], and mirDeep2 [12, 17] require the read count value to be known for predicting miRNAs from RNA-seq data. Although the read count may have good discriminative power [17], it prevents its application to genomic or EST sequences. The presence of the miRNA* can be used by mirDeep2. However, the miRNA* is not always sequenced due to low expression level and degradation [7]. 

Of the three *de novo* predictors of miRNA precursors—NOVOMIR, HHMMiR, and triplet-SVM—NOVOMIR [16] has the highest reported sensitivity and specificity of 83% and 99%, respectively. HHMMiR and triplet-SVM report a sensitivity of 64% and 50%, respectively, for miRNA precursors only. NOVOMIR was trained using *Arabidopsis thaliana* miRNA for positive controls and uses tRNA, rRNA, noncoding RNA, mRNA, and genomic sequences from *A. thaliana* as negative controls. The program reports the predicted precursor along with the start and end positions of any predicted mature miRNA. 

One can ask, what data are necessary to correctly predict miRNAs from sequences? And, specifically, for deep sequencing data, can an accurate predictor be developed that does not require read count or rely on the presence of the miRNA*? The predictive model described here is one of the most specific predictors (98.53% specificity), yet has the least requirements as it only requires that the candidate sequence occurs in a sequence region large enough to encompass the putative precursor. Fulfilling this single requirement allows the set of quantitative sequence properties (attributes) for the candidate sequence to be calculated and passed to the predictor.

Here, positive and negative controls from 18 plant species were used for training and model evaluation. Positive controls were taken from miRBase [18] while negative controls were taken from EST sequences of each species. The negative controls were collected based on the number and length of the known miRNAs in the positive controls for corresponding species. We chose randomly picked ESTs over specific subsets, such as mRNA or various classes of noncoding RNAs, to ensure that the predictor is not accidentally trained to detect the characteristics of the negative controls. Although this would increase sensitivity and specificity of the predictor, it would also introduce bias. ESTs broadly represent protein-coding and nonprotein-coding genes including sequences resembling degraded mRNA fragments that could exist in deep sequencing data [17, 19, 20]. Also, stem-loop structures are commonly found in mRNA. The probability of picking a true miRNA from EST data by random is negligible.

Wet-lab validation is costly and time consuming; therefore, accurately predicting that the candidate RNA is truly a miRNA is important. Our aim was to accurately predict when a sequence is not a miRNA at the expense of missing a few true miRNAs, which limits the number of false positives. To demonstrate the quality of any predictor, rigorous statistical testing is required.

## 2. Methods

### 2.1. Attributes

**Figure 1:**
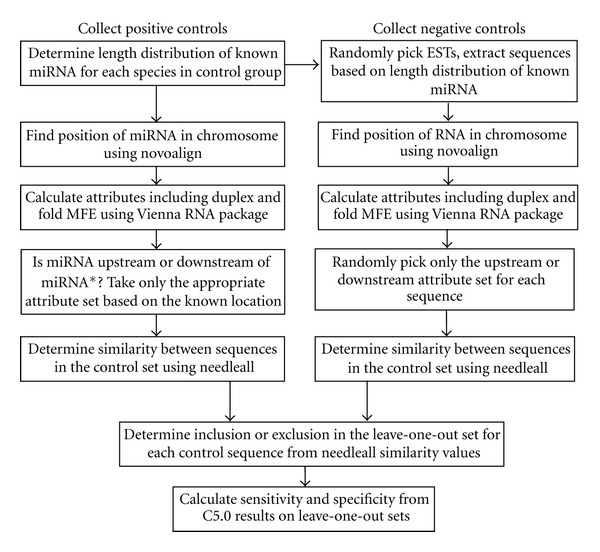
Data processing flow chart for collection of controls, training, and statistical validation. Known miRNAs from miRBase are used as positive controls. Negative controls are randomly picked short segments from ESTs based on the quantity and length distribution of known miRNAs from each plant species in the test set. All controls are aligned to their respective genome. Alignment location allows collection of attributes. For positive controls, the alignment position also allows the location of the miRNA in relation to upstream or downstream of miRNA* to be determined within the precursor. Only attributes from the correct location are valid for training positive controls. Upstream or downstream attributes are equally valid for negative controls, therefore only one is selected at random. Positive controls were aligned using needleall to determine the similarity between all miRNAs. Negative controls were also aligned. The similarity values from the alignment were used to determine if other highly similar sequences should also be excluded when the one in the leave-one-out set was excluded from training. Each sequence in a leave-one-out set was tested for correct classification by the model just trained using controls in the inclusion set. Counts of correct and incorrect classification were used to calculate sensitivity and specificity.

Training of the predictive model requires controls of known outcome. The measured characteristics of the controls used in machine learning are called attributes. The positive controls are known miRNAs and non-miRNA sequences form the negative controls. Known miRNAs from 18 plant species were taken from miRBase Release 18. The negative controls were picked from ESTs of the respective species downloaded from TIGR Plant Transcript Assemblies [21].  Table 1 lists the 18 species by taxonomic group, and Figure 1 is a flow chart of data collection and statistical validation. As stated above, the formation of a hair-pin or stem-loop structure by the precursor is critical for miRNA biogenesis. To obtain attributes for positive controls, the location of the known miRNAs within the precursor stem loop is required. 

**Figure 2:**
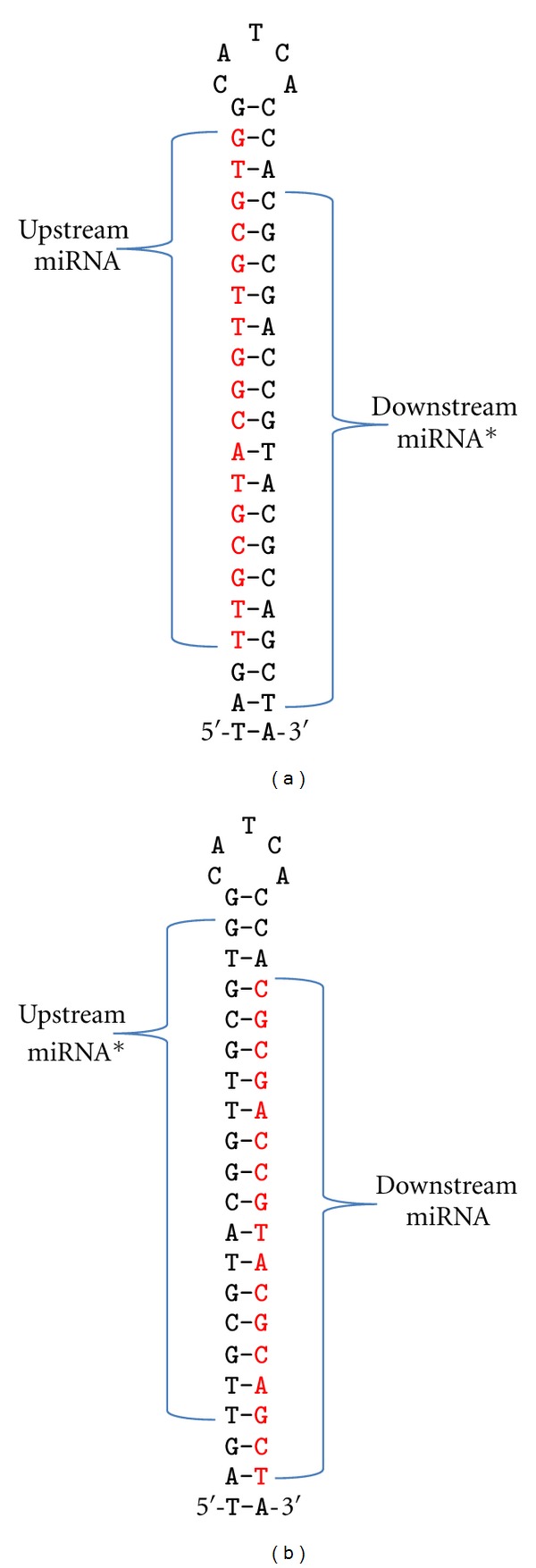
(a) Example of an upstream miRNA with downstream miRNA*. (b) Example of a downstream miRNA with upstream miRNA*. The mature miRNA (in red) may exist within the precursor upstream of the miRNA* (a) or downstream (b). Determining this location is critical for collecting the appropriate attribute set.

The known precursors from miRBase are only used to confirm two things about the miRNAs used as positive controls: (i) the miRNA location on the chromosome is inside a known precursor and not just a random match and (ii) the sequence is upstream or downstream of the miRNA* (in the 5′ half or the 3′ half of the precursor illustrated in Figures 2(a) and 2(b)). Only attributes of miRNAs for these correct chromosome locations and positions in the precursor are included as positive controls. Novoalign [22] was used to align miRNA and precursor sequences to the respective genomes downloaded from PlantGDB [23]. At this point, the known precursors are no longer useful because all sequences will be treated equally from this point on regardless of whether they are positive controls, negative controls, or the assay sequences yet to be classified. 

For any short sequence from the above three types, the precursor candidate was defined by first locating the miRNA* candidate with the strongest duplex binding. Next, the region between and including the sequence and the miRNA* candidate, along with 15 nt on both sides, was extracted. This region defines the operative precursor region (OPR), which in real miRNAs should form a stem-loop structure. The search for the miRNA:miRNA* candidate duplex with the strongest binding was limited to a 300 nt window. The 300 nt window was used because 95.80% of known plant miRNA precursors are less than 300 nt (Table 2). For positive controls we have already defined that the correct window is upstream or downstream of the known miRNA. For negative controls, both orientations are valid. After training, to use the model for prediction on sequences of unknown class, attributes from both upstream and downstream need to be collected and evaluated. This is essentially the same as for negative controls but without prior knowledge of prediction outcome.

**Figure 3:**
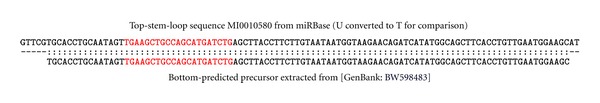
A comparison between precursors from miRBase and the corresponding OPR predicted from the EST data. miRNA lja-miR167 MIMAT0010087 (in red) is found in precursor MI0010580. No genome is listed in miRBase for this miRNA, and it does not align to any chromosome for that species. It does, however, align to EST [GenBank: BW598483]. The EST lja-miR167 is correctly classified as a miRNA with a predicted precursor highly similar to the one in miRBase.

We use the OPR instead of the precursor region as reported by miRBase to ensure equal treatment of all controls, and later for sequences of unknown class. Simply, as unknowns and negative controls do not come with precursors, we must define the OPR equally for all, including the positive controls for training.  Figure 3 illustrates the relationship of the known precursor from miRBase to the OPR defined using the method described above.

The computationally estimated MFE (in kcal/mol) for both the miRNA:miRNA* duplex and the OPR structure are required attributes for training. The RNAduplex function from the Vienna RNA package [24] was used to find the location of the miRNA* and the binding energy of a miRNA:miRNA* duplex within the longer genomic sequences. With this information, the MFE for the OPR can be estimated. RNAfold from the Vienna RNA package was used to calculate the MFE for the OPR, which is represented by *DeltaG* in Table 3. The attribute *DeltaGnorm* is the *DeltaG* normalized to the length of the OPR. RNAfold also returns the secondary structure in dot-bracket format, representing the mismatch and base-pair matching, respectively, for the OPR secondary structure. The dot-bracket structure allows the longest run of uninterrupted matches and mismatches to be calculated, as well as the number of unpaired nucleotides that form the head of the loop. All are normalized to the length of the OPR. These attributes are named *longestDotSet*, *longestBracketSet,* and *loopCountNorm* in Table 3. All other attributes are based on characteristics of the miRNA sequence, for example, the miRNA base composition and the miRNA:miRNA* duplex measurements. The RNAduplex function returns the number of matches and mismatches along with the duplex MFE. It has previously been reported that the number of matching and mismatching base pairs within the precursor stem loop are important attributes for training the precursor predictors [13, 14]. The miRNAs:miRNAs* duplex information that includes base-pair matching and mismatching are also valuable attributes for predicting mature miRNAs.

The attribute *DuplexEnergyNorm* in Table 3 is the MFE of the miRNAs:miRNAs* duplex normalized to the duplex length. The maximum number of consecutive mismatches in the duplex is held in *MaxMismatch*. The minimum number of consecutive matches for each duplex is stored in the attribute *minMatchPercent* and is based on both sides of the duplex depending on the shortest side.  Table 4 lists additional attributes arising from combining base attributes. 

Negative controls are short sequences, randomly picked from the central regions of ESTs. Attributes for negative controls were collected in a similar way as those for positive controls. Sequence lengths were chosen to resemble the length distribution of the positive controls. The randomly picked negative controls only qualified if they contained no ambiguity codes and had a length-normalized Shannon entropy consistent with that of the positive controls. The latter is used to avoid low complexity regions and to ensure that strong negative controls are collected. The attribute *ShannonEntropyNorm* in Table 3 is the Shannon entropy normalized by the sequence length. The negative control sequences were aligned to chromosomes using novoalign. As not all sequences could be aligned (e.g., they may fall across an intron-exon boundary) sufficient quantities were collected to make up for this expected loss. For negative controls, it does not matter whether the operational miRNA is upstream or downstream of the miRNA* as both orientations are equally valid and either the upstream or downstream attribute set is chosen randomly. In this way, the sequence is represented only once in that control set. The final training set used for validation contains 2073 positive controls and 5306 negative controls. More negative controls than positive were used so that the broadest set of non-miRNAs is used to train against the known miRNAs. This increases the model's ability to accurately recognize non-miRNAs and to minimize false positive predictions.

### 2.2. Validation by Leave-One-Out Testing

Once attributes have been collected for both positive and negative controls, validation sets can be produced. The model was validated by calculating sensitivity and specificity based on leave-one-out cross-validation [25]. Sensitivity is the ability of the classifier to identify positive results (true miRNAs), while specificity is the ability to distinguish negative sequences [26]. Leave-one-out cross-validation can be described as putting all controls in a stack, taking the first one out and training with the rest. The predictor just trained is applied to the one excluded from training. Only one of two possibilities will occur: the predictor is correct on the previously unseen control, or not. The one previously removed is returned, and the next one is removed, and again training and testing are done until each control has been excluded and tested. When this is completed, the results are used to calculate sensitivity and specificity based on the following formulas:

(1) $$\begin{array}{c} \text{Sensitivity}=\frac{\text{number}\;\text{of}\;\text{True}\;\text{Positives}}{\text{number}\;\text{of}\;\text{True}\;\text{Positives}+\text{number}\;\text{of}\;\text{False}\;\text{Negatives}}100,\\ \text{Specificity}=\frac{\text{number}\;\text{of}\;\text{True}\;\text{Negatives}}{\text{number}\;\text{of}\;\text{True}\;\text{Negatives}+\text{number}\;\text{of}\;\text{False}\;\text{Positives}}100. \end{array}$$

Only sequences that are not highly similar (based on a cutoff value, see below) to the test sequence will be allowed into the training set. 

MiRNAs found in different locations may be similar or even identical to others. It is important that sequences similar to the ones left out are also excluded to ensure the rigour of the leave-one-out approach. A study on precursor prediction used the BLASTclust program to identify sequences of high similarity for exclusion from the training set [14]. However, we found that BLASTclust does not always ensure that sequences in two different clusters are sufficiently different, as any given sequence can only belong to one cluster. We aligned each sequence to every other sequence and used their similarity to determine inclusion or exclusion from training. In this way, each control sequence is excluded from training together with similar sequences. All nonsimilar sequences are used to train, and this trained model is tested for its ability to accurately predict the class for the one excluded test case. Pools of positive and negative controls have each been pairwise aligned using the needleall program of the EMBOSS package [27]. The results from needleall were used to determine the level of similarity between sequences in the positive controls set, then separately for negative controls. Needleall returns the similarity between two sequences, as well as other values. In this case, two sequences are considered similar if the *similarity* calculated by needleall is greater than 70%, based on the default setting.

### 2.3. Training the Predictor

The C5.0 program from RuleQuest incorporates a decision-tree machine learning method that we have used to train a miRNA predictor using controls of known outcome. C5.0 and the Windows version See5 are improved versions of C4.5 that in turn descended from a program called ID3. The training data used by C5.0 can be any combination of nominal attributes (e.g., the letter of the first nucleotide in the sequence) and numeric attributes (e.g., MFE). The data is evaluated for patterns that discriminate between the training classes. The output is a model in the form of if-then rules or decision trees for classifying cases of unknown outcome. The emphasis is on producing models that are accurate and easy to understand. Producing models that are easy to understand can be useful when the goal is to discover the biological relationships between the attributes and class, rather than to classify unknown cases. In some investigations the goal is to determine the attributes and cutoff values critical for separating the classes. C5.0 can be applied to training data containing many thousands of cases with hundreds of attributes each. The typical size of our training set is ~5294 cases, each with only 29 attributes.

The misclassification cost can also be adjusted when training. It is relatively expensive to validate a miRNA in the wet lab. Our aim is to train a classifier with a low false positive rate. If, after training, the model had a higher than acceptable false positive rate, a misclassification cost would be applied when retraining. If the opposite was true (i.e., validation cheap and miRNA rare), the misclassification cost could be adjusted accordingly. For example, a misclassification cost would be applied to avoid missing true miRNAs during training.

C5.0 supports a technique called boosting [28] that is used to improve classification results. Boosting combines decision trees to produce and test new trees that may improve classification. For example, the branches from one tree are swapped with those from another tree, and the two new classifiers are tested for increased predictive power. Different boosting values were tested to determine the level of improvement to the basic predictor. Figure S1 (see Supplementary Material available online at doi: 10.1155/2012/652979) is a graph of ROC space for 28 runs from 3 to 30 boosting trials. This shows that, for this type of data, lower boosting values already produce good sensitivity and specificity, while higher boosting provides little improvement. An example of a decision tree from training is shown in Figure S2. RuleQuest also provides an evaluator program that uses C5.0-trained classifiers to predict the outcome for data of unknown class. We used this to record the predicted class for each case (e.g., miRNA candidate) along with the confidence value. 

## 3. Results and Discussion

After training, the attribute usage information demonstrates the discriminative importance of each attribute.  Table 5 shows the attribute usage for one of many possible training runs of the classifier; other training runs show similar usage. The values represent the percentage of sequences that required that attribute for classification. Several attributes, such as *DuplexEnergy, minMatchPercent*, and GC content, are required for all sequences to be classified. The G% and C% contents are directly related to the stability of the duplex. Genomic sequences and ESTs from cDNA contain the base T, whereas miRNAs from miRBase contain U. For this work, U was converted to T for processing attributes. The attribute *T%* relates directly to the potential for G-U and A-U base pairing in the RNA. Not surprisingly, the attribute named *DuplexEnergy* is important for training because it relates directly to the stability of the base pairing between the miRNA and miRNA* duplex. This is evident by the 100% usage of *DuplexEnergy* and *DeltaGnorm* which represents the energy of the OPR stem-loop structure normalized by its length. 

Excluding an attribute from training reveals the discriminative importance to the classifier. Although this is an imperfect method, collecting data from all leave-one-out training runs can provide an overall view of an attribute's discriminative power. C5.0 has a built-in function called winnowing that, when applied during training, returns the percentage of increase in error if an attribute is removed from training.  Table 6 shows attributes with the largest average percentage of decline and therefore the level of importance for training the predictor.

Sensitivity and specificity are critical values for assessing classifier accuracy; values as high as 84.08% for sensitivity and 98.53% for specificity have been obtained. The question remains whether this high specificity and sensitivity can also be achieved when the predictor is trained with different species than are used for prediction.

### 3.1. Exclusion and Testing by Taxonomic Group

If all miRNAs in each taxonomic category listed in Table 7 are systematically excluded from training while including all others, how well does the predictor do when tested on the excluded category? Results from these tests reveal how well the model predicts miRNAs in plant species not included in the training set. The ability of the classifier to correctly identify known miRNAs ranged from 78% for the dicotyledon Salicaceae to 100% for 7 of the 18 groups in Table 7. All misclassified miRNAs in the Salicaceae are unique to the single species of poplar tree. In Table 8, four taxonomic groups are formed: monocotyledon, dicotyledon, Lycopodiophyta, and Embryophyta. When Embryophyta is excluded from training, the predictor recognizes 93% of known Embryophyta miRNAs, and 100% of Lycopodiophyta miRNAs when Lycopodiophyta is excluded. In contrast, when Lycopodiophyta, Embryophyta, and one of monocotyledon or dicotyledon are combined, the excluded set of monocotyledon or dicotyledon is recognized at 100%. When Lycopodiophyta and Embryophyta are combined into one group named “primitive,” training with the combination of any two groups produced a model that correctly classifies all miRNAs in the excluded group, as shown in Table 9. For all exclusion experiments, negative controls were correctly classified consistently between 98% and 99%. This demonstrates that an accurate universal predictor of plant miRNAs was produced when all groups, and therefore all miRNAs, were combined for training.

Identical miRNA sequences are found in multiple plant species, often on multiple chromosomes where they are part of identical or different precursors. Although the miRNA sequences can be identical, some of their attributes are not. At a minimum, the location within the chromosome is different. Leave-one-out sets are required for statistical validation by modelling the predictive accuracy of a control sequence that is unseen during training. For validation, excluding identical or near-identical miRNAs is critical. When creating a predictor for unknown sequences, the classifier can be trained using all plant miRNAs to ensure the best predictor possible. The taxonomic exclusion tests demonstrate that training with the maximum number of known miRNAs produces a classifier with the best cross-species recognition of miRNAs. In one training example, the set included 2278 positive controls, some of which are the same miRNAs sequence but in different precursors and species. The contrasting negative controls have 5680 short sequences randomly picked from ESTs. For this training set, from 3 to 300 boosting trials were run. At boosting trials near 300, only five negative controls were misclassified as miRNAs during training. Although only wet-lab validation can confirm that a sequence is a miRNA, two of the five are found in what look like very obvious miRNA precursors. The possible miRNA precursors for these two EST segments are shown in Figures S3 and S4 of the supplementary data. They were classified as miRNAs despite being used as negative controls during training. The folding patterns were produced using QuikFold from the UNAFold Web Server [29]. These two short sequences taken from the middle of randomly picked ESTs have yet to be validated as true miRNAs.

### 3.2. *De Novo* Prediction of miRNAs

One method of demonstrating that the predictor can be used for *de novo* prediction is to see how many known *Medicago truncatula* (Mt) miRNAs can be accurately predicted from the precursor source. This shows the quality of the predictor when applied to short segments taken from ESTs or chromosomes. For Mt, there are 342 unique miRNAs from miRBase that occur in one or more of the 581 unique Mt precursors. Currently recorded miRNAs for Mt in miRBase are 20–24 nt long. Therefore, short sequence segments were taken from the miRNA precursors for only these lengths. The segments were extracted by a sliding window method starting at the beginning of the sequence, taking 20 nt, moving one base over and again taking 20 nt. This continues until the end where it becomes too short for a full 20 nt segment. This is repeated for lengths from 21 to 24. Attributes are calculated for this set of short sequences, and then predictions are made by the trained classifier. Of the 342 unique miRNAs, 309 are correctly classified as miRNAs by the predictor for a score of 90.35%. This demonstrates that the predictor can be applied to short segments taken from ESTs or genomic data as well as to short reads from RNA-seq data. We are currently working to achieve the speed increases required to analyze entire chromosomes.

In some cases, the known miRNA does not match a location on the genome. Of the 18 plant species used for training, only 17 known miRNAs from seven species did not align to the respective genome. When these miRNAs can be found in an EST, the EST sequence can be used in place of a genomic sequence to collect attributes for prediction. An example of this is lja-miR167 in *Lotus japonica*. There is no genome listed for lja-miR167 in miRBase, and it is not found on any chromosomes tested for that species. It is, however, found in an EST [GenBank: BW598483] at position 43. A comparison between the stem loop from miRBase and our predicted OPR for the miRNA is shown in Figure 3. This demonstrates that when a short test sequence is not found in a genome prediction can still occur if it is found in an EST.

During development, short sequences were randomly picked from ESTs in several collections. These negative controls were later pooled and the predictor applied to determine the number of potential miRNAs in random samples of ESTs. Out of 58,443 sequences, 633 (1.08%) were classified as miRNA. Nine (0.02%) of these had the maximum confidence value of 1.00. As described in the methods section, only one miRNA location (either upstream or downstream of the miRNA*) was kept. It is conceivable that if both were kept, the rate of miRNA detection in randomly picked ESTs could double. If some of these negative controls are true miRNA, the specificity would be higher if they were removed from training. While specificity is already high, this suggests that the predictor is more accurate than the value reported here.

The true sensitivity may also be higher than the reported value. Despite using reported miRNAs from miRBase for training, the model refuses to classify all positive controls as miRNA. Fifty miRNAs across 10 species that were not classified as miRNA were collected in a set. These were treated as unknown, and attributes were collected for reclassification. Twelve miRNAs across four species out of the original 50 were classified as miRNA when attributes from both upstream and downstream were tested. Closer inspection of the differences between attributes for the same miRNA between the two sets revealed that in some cases the relative position of miRNA and miRNA* was different. In these cases the precursor in miRBase was asymmetrical, and the automated location picking script returned the wrong location within the precursor. When the correct location and attributes were tested by the predictor, it was classified as a miRNA. An example is gma-MIR5034 MI0017906, where the miRNA is clearly located in the 3′ end. However, it has 69 bases on its downstream side and 60 bases on the upstream side, putting the miRNA predominantly in the upstream half of the precursor. Although some small set of erroneous attributes based on the wrong locations were included in the training dataset, the classifier correctly classified these as miRNA when given both upstream and downstream attributes. Including attributes from incorrect locations for training produced a lower sensitivity value than if correct locations were used but did not diminish the predictor's ability to correctly classify true miRNA. This demonstrates that the predictor is more accurate than the sensitivity reported.

## 4. Conclusion

We have shown that a highly accurate universal plant miRNA predictor can be produced by machine learning using C5.0. This predictor can be applied to any short sequence that aligns to a precursor candidate in a genome or transcriptome. The source of the sequence for testing can be short reads from deep sequencing, or short segments taken from chromosomes or EST sliding windows. Along with miRNA prediction and prediction confidence level, the putative precursor is also produced ready for folding and visualization. If used to scan a chromosome region, this predictor will reveal areas of high or low miRNA density. If applied to deep sequencing data the predictor reveals how often in a genome the short read exists, how many are predicted miRNAs, and how many unique precursors contain that short-read miRNA.

## Supplementary Material

Supplementary Figure S1: The graph shows the relationship of sensitivity and specificity over 28 boosting runs. The insert in the middle of the graph zooms in to show details. It can be seen that the accuracy improves only little with varying levels of boosting trials.
Supplementary Figure S2: A representative decision tree output from running C5.0. The terminal nodes end with classification of Not_miRNA or class_miRNA. The number in parentheses represents the count of sequences correctly classified terminating at the node. If there are two numbers the second is the count of incorrectly classified cases at that node based on training data of known outcome.
Supplementary Figure S3: Sequence TCCTGGCCTGATTGAGTGGCA (shown pulled out from the stem-loop) starting at position 8 from the 5' end is from Sorghum bicoloras *[*GenBank:CN127271*]* and found at position 57396890 on chromosome 5. Despite randomly picking short sequences from ESTs as negative controls for training, the model does not classify some as negative controls.
Supplementary Figure S4: Sequence TGCAAGCCTGTTGTTGAGCGA (shown pulled out from the stem-loop) starting at position 8 from the 5' end is from Vitis viniferaas *[*GenBank:EE095594*]* found on chromosome 6 at position 1508484. Some negative controls classified as miRNA exist within precursor-like predicted stem-loops.

## Figures and Tables

**Table 1:** Plant species from which miRBase miRNAs were used.

Taxonomic group	Species
Brassicaceae	*Arabidopsis thaliana* *Arabidopsis lyrata *
Caricaceae	*Carica papaya *
Embryophyta	*Physcomitrella patens *
Lycopodiophyta	*Selaginella moellendorffii *
Euphorbiaceae	*Ricinus communis *
Fabaceae	*Medicago truncatula* *Lotus japonica* *Glycine max *
Rutaceae	*Citrus clementine* *Citrus sinensis *
Salicaceae	*Populus trichocarpa *
Solanaceae	*Solanum lycopersic *
Vitaceae	*Vitis vinifera *
Poaceae	*Sorghum bicolor* *Oryza sativa *
Panicoideae	*Zea mays *
Pooideae	*Brachypodium distachyon *

**Table:** (a)

3284	Total count of stem loops
938	Max stem loop length
53	Min stem loop length
153	Average stem loop length
132	Median stem loop length

**Table:** (b)

	Count > 300 nt	Count < 300 nt	Count < 350 nt
Counts	135	3146	3194
%	4.11%	95.80%	97.26%

**Table 3:** Base set of attributes.

Attribute	Description of the attribute in relation to the control or candidate sequence
chromLen/position	The ratio of the length of the chromosome over the position on that chromosome
ShannonEntropyNorm	Shannon entropy normalized to the sequence length
G%	Percentage of G base composition
C%	Percentage of C base composition
T%	Percentage of T base composition
A%	Percentage of A base composition
DuplexEnergy	The duplex energy between the miRNAs:miRNAs*
DuplexEnergyNorm	The duplex energy normalized to the length of the duplex structure
MaxMismatch	Maximum number of mismatches in the duplex structure based on both sides of the structure
minMatchPercent	Minimum % match based on length of the duplex structure both sides of the structure
DeltaG	Minimum free energy for the stem loop
DeltaGnorm	Minimum free energy normalized to the length of the stem loop
longestDotSet	Longest run of mismatches in the stem loop
longestBracketSet	Longest run of matches in the stem loop
loopCountNorm	Number of loop heads normalized to the length of the stem loop

**Table 4:** Extended attribute set from combinations of base attributes.

Attribute	Description of the attribute in relation to the control or candidate sequence
G + T:= G% + T%	Sum of G% and T%
G/T:= G%/T%	Ratio from G% to T%
G + C:= G% + C%	Sum of G% and C%
G/C:= G%/C%	Ratio from G% to C%
A + C:= A% + C%	Sum of A% and C%
A/C:= A%/C%	Ratio from A% to C%
T + A:= T% + A%	Sum of T% and A%
T/A:= T%/A%	Ratio from T% to A%
G%/ShannonEntropyNorm := G%/ShannonEntropyNorm	Ratio of G% over normalized Shannon entropy
C%/ShannonEntropyNorm := C%/ShannonEntropyNorm	Ratio of C% over normalized Shannon entropy
T%/ShannonEntropyNorm := T%/ShannonEntropyNorm	Ratio of T% over normalized Shannon entropy
A%/ShannonEntropyNorm := A%/ ShannonEntropyNorm	Ratio of A% over normalized Shannon entropy
NormEnergyRatio := DeltaGnorm/DuplexEnergyNorm	Ratio of the normalized DeltaG from the stem loop and normalized miRNAs:miRNAs* duplex energy
longestBracket/longestDot := longestBracketSet / longestDotSet	Ratio of longest match over the longest mismatch normalized counts

**Table 5:** Example of attribute usage from one representative training run.

Attribute usage	
100%	G%
100%	C%
100%	T%
100%	DuplexEnergy
100%	minMatchPercent
100%	DeltaGnorm
100%	G + T
100%	G + C
98%	duplexEnergyNorm
86%	NormEnergyRatio
85%	MaxMismatch
82%	ShannonEntropyNorm
74%	G/T
51%	A%
51%	A + C
28%	chromLen/position

**Table 6:** Attributes with an average decline of 1% or greater when excluded.

Attribute	Average percentage decline in training accuracy when the attribute is removed
DuplexEnergy	53%
T% + A%	20%
DeltaGnorm	14%
longestBracketSet	10%
minMatchPercent	7%
G% + C%	5%
loopCountNorm	4%
MaxMismatch	4%
DuplexEnergyNorm	1%
DeltaG	1%
longestBracket/longestDot	1%

**Table 7:** Results from exclusion of each of the 13 taxonomic groups.

Taxonomic grouping	Error count	Total count of miRNAs	% correctly classified	% of full set excluded	Notes
Embryophyta	12	190	94	9.16	*Physcomitrella patens* (moss) —model organism
Lycopodiophyta	0	55	100	2.65	*Selaginella moellendorffii* (ancient vascular plant lineage)—model organism
Brassicaceae	0	440	100	20.22	Dicot, two *Arabidopsis *
Caricaceae	0	1	100	0.05	Dicot, papaya
Euphorbiaceae	0	7	100	0.34	Dicot, castor oil plant
Fabaceae	0	560	100	27.00	Dicot, three legumes
Salicaceae	16	73	78	3.52	Dicot, poplar tree
Solanaceae	1	14	93	0.68	Dicot, tomato plant
Vitaceae	9	89	94	4.29	Dicot, common grape
Rutaceae	0	9	100	0.43	Dicot, two citrus trees
Panicoideae	8	166	95	8.00	Monocot, *Zea mays *
Poaceae	0	404	100	19.48	Monocot, rice and sorghum
Pooideae	13	66	80	3.18	Monocot, *Brachypodium distachyon* (model organism)

**Table 8:** Results from exclusion of each of the four taxonomic groups.

Taxonomic grouping	Error count	Total count of miRNAs	% correctly classified	% of full set excluded	Notes
Embryophyta	13	190	93	9.16	*Physcomitrella patens* (moss)—model organism
Lycopodiophyta	0	55	100	2.65	*Selaginella moellendorffii* (ancient vascular plant lineage)—model organism
Monocotyledons	0	1193	100	57.52	Four species
Dicotyledons	0	636	100	30.67	Twelve species

**Table 9:** Results from exclusion of each of the 3 taxonomic groups.

Taxonomic grouping	Error count	Total count of miRNAs	% correctly classified	% of full set excluded	Notes
Primitive	0	389	100	11.81	*Physcomitrella patens* (moss) and *Selaginella moellendorffii* (ancient vascular plant lineage), two model organisms
Monocotyledons	0	1775	100	30.67	Four species
Dicotyledons	0	946	100	57.52	Twelve species
