# Corrigendum to “Plant MicroRNA Prediction by Supervised Machine Learning Using C5.0 Decision Trees”

**DOI:** 10.1155/2017/7876832

**Published:** 2017-10-24

**Authors:** Philip H. Williams, Rodney P. Eyles, Georg Weiller

**Affiliations:** Division of Plant Sciences, Research School of Biology, College of Medicine, Biology & Environment, The Australian National University, Canberra, ACT 0200, Australia

In the article titled “Plant MicroRNA Prediction by Supervised Machine Learning Using C5.0 Decision Trees” [1] the name of the second author was given incorrectly as Rod Eyles. The author's name should have been written as Rodney P. Eyles. The revised authors' list is shown above.
