# Supplementary material for: Plant MicroRNA Prediction by Supervised Machine Learning Using C5.0 Decision Trees
Source: J Nucleic Acids. 2012 Nov 7;2012:652979. doi: 10.1155/2012/652979 (PMC3503367; doi:10.1155/2012/652979)
Supplement: Supplementary file 1 — Supplementary Figure S1: The graph shows the relationship of sensitivity and specificity over 28 boosting runs. The insert in the middle of the graph zooms in to show details. It can be seen that the accuracy improves only little with varying levels of boosting trials. Supplementary Figure S2: A representative decision tree output from running C5.0. The terminal nodes end with classification of Not_miRNA or class_miRNA. The number in parentheses represents the count of sequences correctly classified terminating at the node. If there are two numbers the second is the count of incorrectly classified cases at that node based on training data of known outcome. Supplementary Figure S3: Sequence TCCTGGCCTGATTGAGTGGCA (shown pulled out from the stem-loop) starting at position 8 from the 5' end is from Sorghum bicoloras [GenBank:CN127271] and found at position 57396890 on chromosome 5. Despite randomly picking short sequences from ESTs as negative controls for training, the model does not classify some as negative controls. Supplementary Figure S4: Sequence TGCAAGCCTGTTGTTGAGCGA (shown pulled out from the stem-loop) starting at position 8 from the 5' end is from Vitis viniferaas [GenBank:EE095594] found on chromosome 6 at position 1508484. Some negative controls classified as miRNA exist within precursor-like predicted stem-loops. [file 652979.f1.doc]

## Figure S1. The graph shows the relationship of sensitivity and specificity over 28 boosting runs. The insert in the middle of the graph zooms in to show details. It can be seen that the accuracy improves only little with varying levels of boosting trials.


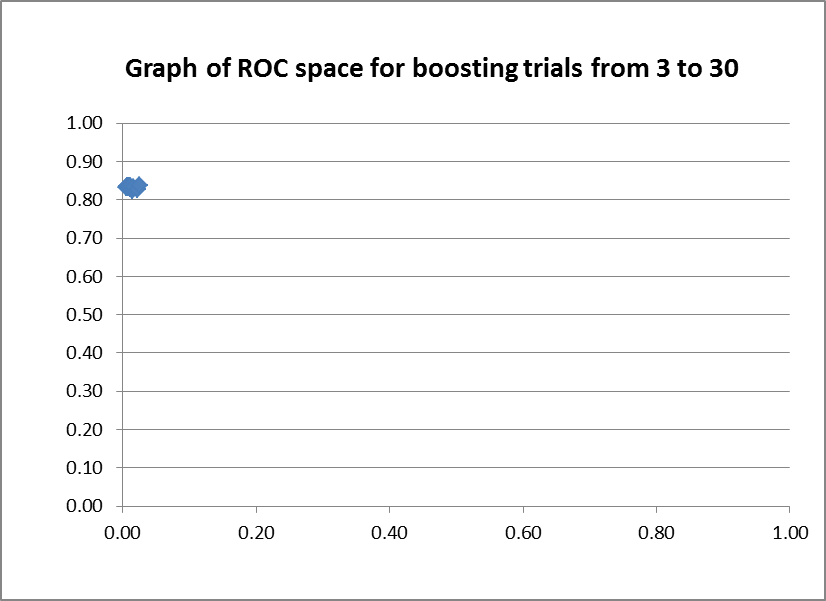


Sensitivity

1-Specificity

## Figure S2. A representative decision tree output from running C5.0. The terminal nodes end with classification of Not_miRNA or class_miRNA. The number in parentheses represents the count of sequences correctly classified terminating at the node. If there are two numbers the second is the count of incorrectly classified cases at that node based on training data of known outcome.

Decision tree:

DuplexEnergy <= -21.2:

:...MaxMismatch > 2:

: :...G+C > 65.22:

: : :...loopCountNorm <= 45.67:

: : : :...LoopLen/DeltaG <= -1.89: Not_miRNA (170/1)

: : : : LoopLen/DeltaG > -1.89:

: : : : :...longestBracket/longestDot <= 0.3753: class_miRNA (3)

: : : : longestBracket/longestDot > 0.3753: Not_miRNA (12/1)

: : : loopCountNorm > 45.67:

: : : :...longestBracketSet > 10.45: Not_miRNA (156/21)

: : : longestBracketSet <= 10.45:

: : : :...T% <= 30.43: class_miRNA (10/1)

: : : T% > 30.43: Not_miRNA (3)

: : G+C <= 65.22:

: : :...longestBracketSet > 17.54:

: : :...DuplexEnergy <= -26.03: class_miRNA (3)

: : : DuplexEnergy > -26.03:

: : : :...loopCountNorm <= 72: Not_miRNA (24)

: : : loopCountNorm > 72: class_miRNA (2)

: : longestBracketSet <= 17.54:

: : :...LoopLen/DeltaG > -2.88: class_miRNA (59/3)

: : LoopLen/DeltaG <= -2.88:

: : :...FirstBaseAttrib = G: Not_miRNA (6)

: : FirstBaseAttrib = A:

: : :...T% <= 33.33: Not_miRNA (2)

: : : T% > 33.33: class_miRNA (2)

: : FirstBaseAttrib = C:

: : :...longestDotSet <= 12.28: Not_miRNA (2)

: : : longestDotSet > 12.28: class_miRNA (5)

: : FirstBaseAttrib = T:

: : :...minMatchPercent <= 73.68: Not_miRNA (3)

: : minMatchPercent > 73.68: class_miRNA (6)

: MaxMismatch <= 2:

: :...G+C <= 72.72:

: :...longestBracketSet > 16.71:

: : :...DuplexEnergy <= -24.66: class_miRNA (73/5)

: : : DuplexEnergy > -24.66:

: : : :...T+A <= 61.9: Not_miRNA (52/15)

: : : T+A > 61.9: class_miRNA (14/2)

: : longestBracketSet <= 16.71:

: : :...minMatchPercent > 80.95: class_miRNA (1267/6)

: : minMatchPercent <= 80.95:

: : :...LoopLen/DeltaG <= -2.9:

: : :...StrucLen/duplexEnergy > -0.73: class_miRNA (6)

: : : StrucLen/duplexEnergy <= -0.73:

: : : :...minMatchPercent <= 80: Not_miRNA (7)

: : : minMatchPercent > 80: class_miRNA (14/6)

: : LoopLen/DeltaG > -2.9:

: : :...chromLen/position <= 23.67: class_miRNA (63/1)

: : chromLen/position > 23.67:

: : :...LoopLen/DeltaG <= -2.02: class_miRNA (3)

: : LoopLen/DeltaG > -2.02: Not_miRNA (3)

: G+C > 72.72:

: :...LoopLen/DeltaG > -1.6: class_miRNA (16)

: LoopLen/DeltaG <= -1.6:

: :...loopCountNorm <= 53.33:

: :...DuplexEnergy > -25.9: Not_miRNA (38)

: : DuplexEnergy <= -25.9:

: : :...T+A <= 43.48: Not_miRNA (41/6)

: : T+A > 43.48: class_miRNA (4)

: loopCountNorm > 53.33:

: :...DeltaG > -66: class_miRNA (28/1)

: DeltaG <= -66:

: :...DuplexEnergy > -28.3: Not_miRNA (21)

: DuplexEnergy <= -28.3:

: :...G+C <= 85: class_miRNA (9)

: G+C > 85: Not_miRNA (6/1)

DuplexEnergy > -21.2:

:...minMatchPercent > 85.71:

:...longestBracketSet <= 14.29:

: :...StrucLen/duplexEnergy > -1.32:

: : :...T+A > 55.56: class_miRNA (99/6)

: : : T+A <= 55.56:

: : : :...DeltaG <= -25.8: Not_miRNA (8)

: : : DeltaG > -25.8: class_miRNA (3)

: : StrucLen/duplexEnergy <= -1.32:

: : :...MaxMismatch <= 1: class_miRNA (17/5)

: : MaxMismatch > 1:

: : :...DuplexEnergy > -13.78: Not_miRNA (16)

: : DuplexEnergy <= -13.78:

: : :...StrucLen/duplexEnergy <= -1.52: class_miRNA (5)

: : StrucLen/duplexEnergy > -1.52:

: : :...longestBracket/longestDot <= 0.4848: class_miRNA (3)

: : longestBracket/longestDot > 0.4848: [S1]

: longestBracketSet > 14.29:

: :...StrucLen/duplexEnergy <= -1.45: Not_miRNA (105/4)

: StrucLen/duplexEnergy > -1.45:

: :...G+C <= 41.67:

: :...NormEnergyRatio <= 3.417: class_miRNA (7)

: : NormEnergyRatio > 3.417: Not_miRNA (2)

: G+C > 41.67:

: :...T+A <= 52.38: Not_miRNA (26)

: T+A > 52.38:

: :...longestBracketSet > 21.67: Not_miRNA (57/4)

: longestBracketSet <= 21.67:

: :...FirstBaseAttrib = T: class_miRNA (16/4)

: FirstBaseAttrib = G: Not_miRNA (12/2)

: FirstBaseAttrib = A:

: :...longestBracketSet <= 16.33: Not_miRNA (4)

: : longestBracketSet > 16.33: class_miRNA (4)

: FirstBaseAttrib = C:

: :...DuplexEnergy <= -18.4: class_miRNA (5/1)

: DuplexEnergy > -18.4: Not_miRNA (7)

minMatchPercent <= 85.71:

:...longestBracketSet > 11.62:

:...DuplexEnergy > -19.1: Not_miRNA (3930/151)

: DuplexEnergy <= -19.1:

: :...longestBracketSet > 15.23: Not_miRNA (245/15)

: longestBracketSet <= 15.23:

: :...T+A > 69.57: class_miRNA (4)

: T+A <= 69.57:

: :...T%/ShannonEntropy <= 4.019: Not_miRNA (32/2)

: T%/ShannonEntropy > 4.019:

: :...minMatchPercent <= 84: Not_miRNA (16/5)

: minMatchPercent > 84: class_miRNA (7/1)

longestBracketSet <= 11.62:

:...minMatchPercent > 80.95:

:...C% > 27.78: Not_miRNA (21/4)

: C% <= 27.78:

: :...loopCountNorm <= 40: Not_miRNA (12/3)

: loopCountNorm > 40: class_miRNA (44/6)

minMatchPercent <= 80.95:

:...loopCountNorm > 45.75:

:...LoopLen/DeltaG <= -2.98: Not_miRNA (102/19)

: LoopLen/DeltaG > -2.98:

: :...T+A <= 52.64: Not_miRNA (5)

: T+A > 52.64: class_miRNA (24/1)

loopCountNorm <= 45.75:

:...StrucLen/duplexEnergy > -0.87:

:...StrucLen/duplexEnergy <= -0.85: class_miRNA (5/1)

: StrucLen/duplexEnergy > -0.85: Not_miRNA (17/3)

StrucLen/duplexEnergy <= -0.87:

:...G/C <= 3.33: Not_miRNA (346/25)

G/C > 3.33:

:...MaxMismatch <= 2: class_miRNA (4)

MaxMismatch > 2:

:...FirstBaseAttrib = C: Not_miRNA (0)

FirstBaseAttrib in {A,G}: Not_miRNA (10/1)

FirstBaseAttrib = T:

:...chromLen/position <= 4.85: class_miRNA (4)

chromLen/position > 4.85: Not_miRNA (2)

SubTree [S1]

longestBracket/longestDot <= 1.633: Not_miRNA (15)

longestBracket/longestDot > 1.633: class_miRNA (2)

##
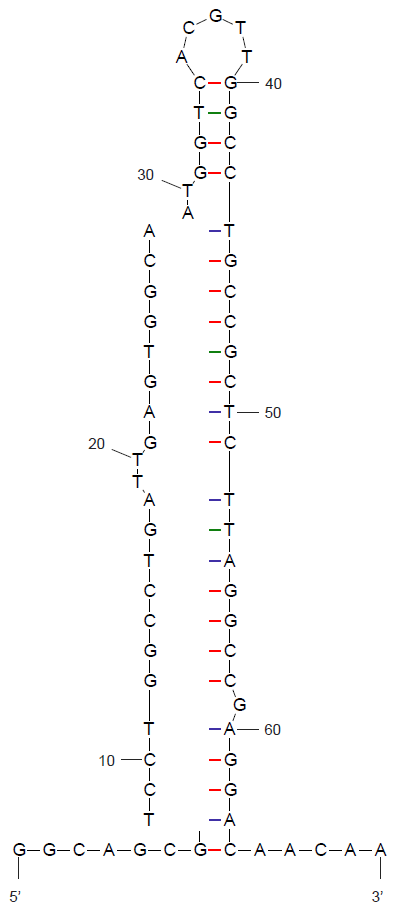
Figure S3 - Sequence TCCTGGCCTGATTGAGTGGCA (shown pulled out from the stem-loop) starting at position 8 from the 5’ end is from *Sorghum bicoloras* [GenBank:CN127271] and found at position 57396890 on chromosome 5. Despite randomly picking short sequences from ESTs as negative controls for training, the model does not classify some as negative controls.

##
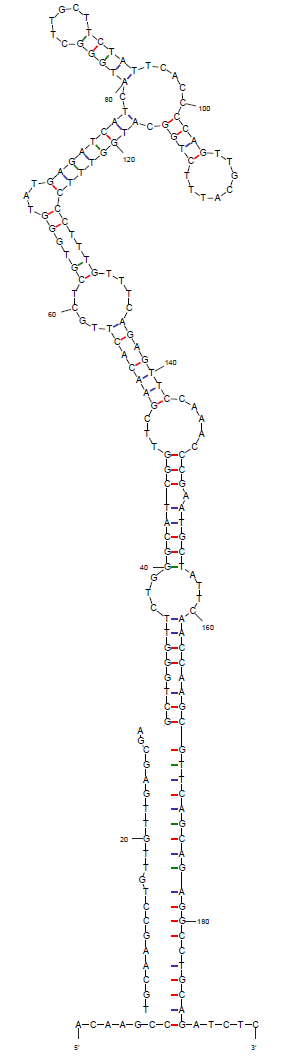
Figure S4 - Sequence TGCAAGCCTGTTGTTGAGCGA (shown pulled out from the stem-loop) starting at position 8 from the 5’ end is from *Vitis viniferaas* [GenBank:EE095594] found on chromosome 6 at position 1508484. Some negative controls classified as miRNA exist within precursor-like predicted stem-loops.
